# Mirvetuximab Soravtansine in Platinum-Resistant Ovarian Cancer

**DOI:** 10.1177/11795549231187264

**Published:** 2023-07-25

**Authors:** Eduardo Gonzalez-Ochoa, Ana C Veneziani, Amit M Oza

**Affiliations:** 1 Division of Medical Oncology and Hematology, Princess Margaret Cancer Centre, University Health Network, Toronto, ON, Canada; 2 Department of Medicine, University of Toronto, Toronto, ON, Canada

**Keywords:** Antibody drug conjugate, folate receptor, mirvetuximab, ovarian cancer, platinum resistance

## Abstract

Ovarian cancer is the second leading cause of death from gynecologic malignancies worldwide. Management of platinum-resistant disease is challenging and clinical outcomes with standard chemotherapy are poor. Over the past decades, significant efforts have been made to understand drug resistance and develop strategies to overcome treatment failure. Antibody drug conjugates (ADCs) are a rapidly growing class of oncologic therapeutics, which combine the ability to target tumor-specific antigens with the cytotoxic effects of chemotherapy. Mirvetuximab soravtansine is an ADC comprising an IgG1 monoclonal antibody against the folate receptor alpha (FRα) conjugated to the cytotoxic maytansinoid effector molecule DM4 that has shown promising clinical activity in patients with FR-α-positive ovarian cancer. This review summarizes current evidence of mirvetuximab soravtansine in platinum-resistant ovarian cancer, focusing on clinical activity, toxicity, and future directions.

## Introduction

Ovarian cancer is the second leading cause of death from gynecologic malignancies worldwide, with an estimated 207 252 deaths from the disease in 2020.^1^ Epithelial ovarian cancer (EOC) accounts for over 95% of ovarian malignancies, of which high-grade serous ovarian cancer (HGSOC) is the most common histologic subtype with 70% of cases.^2^ It is characterized by severe genomic instability, with nearly universal *TP53* mutations, and defects in homologous recombination DNA repair pathways in half of the cases.^3^

Patients are usually diagnosed with advanced disease (FIGO stages III/IV) and standard treatment consists of debulking surgery followed by adjuvant platinum doublet chemotherapy. Since its introduction in the 1960s, platinum has remained the mainstay of systemic therapy in this disease. Despite high initial response rates, up to 80% of patients relapse and, unfortunately, ultimately die from the disease.^4^

Recurrent ovarian cancer has been classified into 4 subgroups based on the platinum-free interval (PFI), defined as the time from the last dose of platinum to disease progression: platinum-refractory (<1 month), platinum-resistant (1-6 months), partially platinum-sensitive (6-12 months), and fully platinum-sensitive (>12 months).^5^ PFI was arbitrarily defined based on observational studies and a probabilistic partition with the likelihood of response being a continuous variable^6^; this definition has been used in clinical trials to define eligibility for retreatment with platinum upon disease progression. More recently, the 6th Ovarian Cancer Consensus Conference proposed to replace PFI with the term treatment-free interval (TFI), dividing it into platinum-TFI (TFIp), nonplatinum-TFI (TFInp), and biologic agent-TFI (TFIb) to better define different trial populations and recommended that patients who had relapsed within 12 weeks of their last platinum dose be selected for a next line of therapy that excludes platinum.^7^

PFI is a key prognostic factor in recurrent EOC. Patients with platinum-sensitive disease have a higher likelihood of responding to additional platinum-based therapy and an overall survival expectation of around 2 to 3 years^8^; however, virtually all patients will eventually develop acquired or secondary resistance. Patients with platinum-resistant disease have a poor prognosis and are usually treated with sequential monochemotherapies, including weekly Paclitaxel, liposomal Doxorubicin, Topotecan, and Gemcitabine, with low response rates of around 10% to 20%, and a survival expectation of less than 12 months.^9,10^

Platinum resistance is a continuum, multifactorial process, and biological mechanisms remain to be fully elucidated.^11^ Over the past decades, significant efforts have been made to understand the sensitivity to therapy, as well as intrinsic or acquired resistance, and to evaluate strategies to overcome treatment failure.

Targeted agents have been gradually introduced into clinical practice for the treatment of recurrent disease, and in the last 10 years, 2 different classes of drugs have been approved.

Angiogenesis inhibitor Bevacizumab has been shown to improve clinical outcomes when added to chemotherapy in initial therapy, and following recurrence as concurrent and maintenance treatment.^12-14^ Poly (ADP-ribose) polymerase inhibitors (PARPi) Olaparib, Niraparib, and Rucaparib were initially approved in the recurrent setting and have moved earlier in the treatment paradigm.^15^ Current standard of care incorporates bevacizumab and PARPi therapy in conjunction with platinum doublet chemotherapy, with concurrent and maintenance approaches, informed by the clinical assessment of risk (residual disease), molecular assessment of BRCA mutational status, and quantitative assessment of homologous recombination deficiency (HRD).

Antibody drug conjugates (ADCs) are a rapidly growing class of oncologic therapeutics, which combine the ability to target tumor-specific antigens with the cytotoxic effects of chemotherapy.

## Antibody Drug Conjugates

ADCs are complex engineered molecules that consist of a monoclonal antibody (mAb) directed toward tumor-associated antigens, conjugated via a stable linker to a potent cytotoxic agent.^16^ Each component contributes to an ADC’s biodistribution, tumor specificity, and cytotoxic effects. The primary goal of ADCs is to improve the therapeutic index of antineoplastic agents by restricting their systemic delivery to cells that express the target antigen of interest.^17^

The selection of the antigen to which the ADC will bind is crucial. The antigen confers specificity of the ADC, thus should be highly or preferentially expressed on the tumor cell, minimally expressed on normal tissues, and present on the cell surface to allow recognition and binding by the circulating ADC; it should also possess internalization properties as it will facilitate the ADC to transport into the cell, which will in turn enhance the efficacy of the cytotoxic agent.^18,19^

There are currently 6 FDA-approved ADCs in solid (nonhematologic) tumors: Trastuzumab emtansine (anti-Her2 and maytansinoid DM1 conjugate) and Trastuzumab deruxtecan (anti-Her2 and camptothecin DXd conjugate) for Her2-positive metastatic breast cancer; Enfortumab vedotin (anti-Nectin4 and auristatin MMAE conjugate) for metastatic urothelial cancer; Sacituzumab govitecan (anti-Trop2 and camptothecin SN-38 conjugate) for triple-negative metastatic breast cancer; Tisotumab vedotin (antitissue factor and auristatin MMAE conjugate) for recurrent/metastatic cervical cancer; and Mirvetuximab soravtansine (antifolate receptor alpha, and maytansinoid DM4 conjugate) for platinum-resistant ovarian cancer, the focus of this review.

## Targeting the Folate Receptor Alpha (FRα)

Folates are important one-carbon donors for the synthesis of purines and thymidine, essential components of nucleic acids, and for the methylation of DNA, proteins, and lipids.^20^ In adult tissues, folate is mainly taken up by a reduced folate carrier, a ubiquitously expressed anion channel that has a relatively low folate-binding affinity.^21^ By contrast, high-affinity uptake of the food supplement folic acid and the physiologically prevalent folate N5-methyltetrahydrofolate (5-mTHF) requires the function of 3 subtypes of folate receptors (FRα, FRβ, and FRγ), which are cysteine-rich glycoproteins that mediate folate uptake through endocytosis.^22,23^ Among the 3 isoforms, FRα is the most widely expressed,

FRα is a 38 to 40 kDa glycosyl-phosphatidylinositol (GPI)-anchored cell-surface glycoprotein encoded by the FOLR1 gene.^24^ It has a scarce distribution across several nonmalignant tissues of the choroid plexus, thyroid, salivary glands, breast, colon, and bladder.^25^ In contrast, FRα overexpression is characteristic of a number of epithelial tumors, including ovarian, endometrial, triple-negative breast, and non-small-cell lung cancers (NSCLC).^26^

Up to 90% of ovarian cancers constitutively express FRα, which is minimal in the normal ovarian epithelium.^27-29^

High FRα expression has been associated with poorly differentiated, more aggressive tumors, as well as resistance to conventional chemotherapy.^30,31^ FRα expression has been preserved over time and not affected by prior therapy exposure.^32,33^ In a phase I, expansion study (NCT01609556), the concordance of FRα expression in archival and biopsy tissues from 21 patients with relapsed EOC was 71%, and no major shifts in receptor expression were observed in matched pretreatment and posttreatment biopsy samples. In addition, higher FRα expression (regardless of the tissue source analyzed) was associated with greater antitumor activity.^33^ Thus, FRα emerged as a potential candidate for molecularly targeted approaches.^34^

The development of FRα-selective therapies requires an accurate quantification of tumor FRα expression, to use this measure as a response-predictive biomarker for patient selection.^29^ Adequate selection will depend on the efficiency of detecting FRα, and clinical trials should incorporate stratification based on receptor status and need to confirm the clinical behavior of high vs low expressers, hence need a control cohort in the same population.

## Early Approaches Targeting the Folate Receptor

The initial clinical evaluation of the first folate receptor-targeting agents provided critical proof-of-concept evidence for FRα as a druggable target for cancer treatment.

Farletuzumab (MORAb-003), a nonconjugated humanized IgG1 antibody, was one of the first FRα-targeted agents evaluated in the clinic. It promotes cell death via antibody-dependent cellular cytotoxicity and complement-dependent cytotoxicity.^35^ A phase II study in patients with platinum-sensitive ovarian cancer showed an overall response rate (ORR) of 75% in patients treated with farletuzumab, carboplatin and taxane, followed by farletuzumab maintenance.^36^ Nevertheless, a phase III trial evaluating carboplatin and taxane plus farletuzumab or placebo did not meet its primary endpoint of improved progression-free survival in the platinum-sensitive setting.^37^ A second phase III trial with Farletuzumab plus paclitaxel in patients with platinum-resistant ovarian cancer (NCT00738699) was stopped early due to futility. A lack of a prior patient selection based on the FRα expression level has been suggested to be a contributing factor to the failure of these studies.^38^

Vintafolide (EC145) is a small-molecule folate-cytotoxic agent linked through a peptide spacer to the microtubule-destabilizing agent desacetylvinblastine monohydrazide.^39^ The phase II PRECEDENT trial evaluated Vintafolide in combination with pegylated liposomal doxorubicin (PLD) using an imaging agent (Etarfolatide) that allows single photon emission computed tomography (SPECT) imaging of FR-expressing tumors. The combination demonstrated a progression-free survival (PFS) benefit compared with PLD plus placebo (5.0 vs 2.7 months, *P* = .031) in patients with platinum-resistant ovarian cancer.^40^ The greatest benefit was seen in patients whose tumors were 100% positive for FRα expression. However, the phase III PROCEED study failed to confirm prior results, as it was stopped at interim analysis due to futility.^41^

## Mirvetuximab Soravtansine

Mirvetuximab soravtansine (MIRV, IMGN853) is an ADC comprising a humanized FRα-binding monoclonal IgG1 antibody (M9346A) conjugated to the cytotoxic maytansinoid effector molecule DM4 through a charged, cleavable disulfide linker.^42,43^ M9346A was selected from a panel of murine anti-FRα antibodies, optimized based on the ability to deliver a maytansinoid payload to FRα-positive cells, and then humanized by variable domain resurfacing.^44^ Further evaluation of M9346A conjugates demonstrated that conjugation of DM4 with the linker sulfo-SPDB [*N*-succinimydl 4-(2-pyridyldithio)-2-sulfobutanoate] provided the highest activity in FRα-expressing xenograft tumor models and was finally incorporated to the final ADC molecule.^44,45^

**Figure 1.**
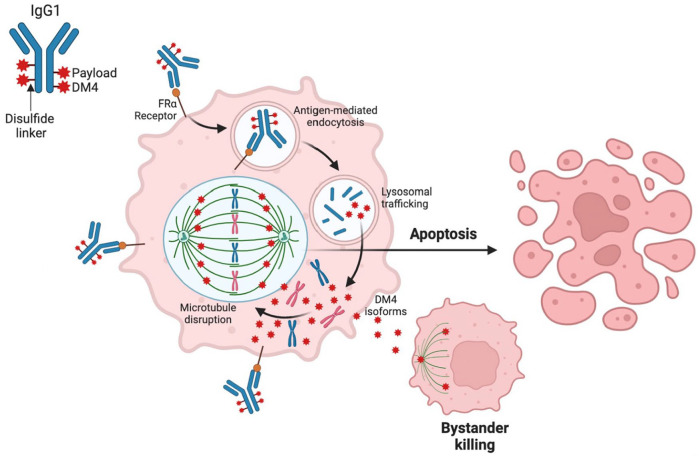
Mirvetuximab soravtansine mechanism of action.

MIRV is conjugated with a drug-to-antibody ratio (DAR) of 3.5:1. It binds with high affinity and specificity to FRα on the surface of the tumor cells, which, upon antigen binding, promotes ADC internalization via antigen-mediated endocytosis, delivered to lysosomes by vesicular trafficking and then degraded to 3 forms of the payload: lysine-Nεsulfo-SPDB-DM4, S-methyl-DM4, and DM4, all of which can inhibit tubulin polymerization and disrupt microtubule assembly, resulting in G2-M arrest and apoptosis.^45-47^ In addition, S-methylDM4 is electrically neutral and lipophilic, thus able to diffuse across biomembranes into proximal tumor cells and kill them, an effect known as bystander killing^48^ (Figure 1).

## Preclinical Activity

Preclinical characterization of MIRV demonstrated that it reduced viability in FRα-positive tumor cells in vitro with low nanomolar potency, and the level of expression of FRα on the surface of cells was found to be a major determinant in the sensitivity of tumor cells to the cytotoxic effect of the conjugate.^44^

Antitumor activity was tested in vivo in mice bearing ovarian cancer cell line-derived xenografts with variable FRα expressions. MIRV was found to be highly active in all FRα-positive xenograft models causing either complete or partial regressions at the highest dose tested (approximately 5 mg/kg). The conjugate was inactive against the FRα-negative tumors, and a control conjugate of a nontargeting isotype-matched antibody was also inactive, demonstrating that MIRV activity is FRα-selective. The conjugate was well tolerated at all tested doses, and no toxicity was observed after treatment.^44^

Combination of MIRV with carboplatin or doxorubicin resulted in synergistic antiproliferative effects in vitro via cell-cycle arrest and augmented DNA damage, and translated into improved antitumor activity in patient-derived xenograft models in vivo.^49^ MIRV also improved the in vivo efficacy of Bevacizumab in platinum-resistant EOC models, causing significant regressions and complete responses in the majority of tumor-bearing mice.^49^

## Pharmacokinetics

In the first human phase I study, after the first administration of MIRV, mean exposure (maximum plasma concentration [C_max_] and area under the concentration-time curve from zero to infinity [AUC0-∞]) increased proportionally with doses from 1.0 to 7.0 mg/kg. Mean half-life (t1/2) for MIRV ranged from 79 to 121 hours across doses, with no meaningful dose-dependence noted in total clearance or volume of distribution for patients that received ⩾1.0 mg/kg; exposure metrics on subsequent cycles showed no meaningful accumulation of MIRV after multiple doses.^50^

## Clinical Activity

In 2017, Moore and colleagues published the results of the first human, phase I study of MIRV in advanced solid tumors (IMGN853-0401), including ovarian, endometrial, renal, and non-small-cell lung cancer (NSCLC), not selected by folate receptor expression.^50^ The recommended phase 2 dose was established on 6 mg/kg; the most common side effects included fatigue, blurred vision, diarrhea, and neuropathy. The expansion phase included 46 patients with platinum-resistant ovarian cancer (PROC) and up to five prior lines of systemic therapy, with FRα-positive tumors, defined as ⩾25% of tumor cells with at least 2 + stain intensity on immunohistochemistry (IHC).^51^ Among 46 patients, the overall response rate was 26%, median DOR of 19.1 weeks and PFS of 4.8 months (Table 1). In this study, the majority of patients experienced response, regardless of FRα expression (low = 25%-49% of tumor cells with ⩾2 + intensity; medium = 50%-74%; high ⩾ 75%). Of note, in patients who received less than three prior lines of therapy, ORR was 39%.^51^ In comparison, response rates to single-agent agent chemotherapy with weekly Paclitaxel, PLD, Topotecan, or Gemcitabine in this setting are around 10% to 30%.^52-54^

**Table 1.** Efficacy of MIRV in FRα-positive platinum-resistant ovarian cancer.

	**FRα score**	**ORR, %**		**mPFS, mo**		**mOS, mo**	
		All patients	FRα high	All patients	FRα high	All patients	FRα high
**IMGN853-0401** *Phase I (n* *=* *46)*^51^	PS2+	26	26	4.8	NA	NA	NA
**FORWARD I** MIRV vs ICC *Phase III (n* *=* *243)*^55,56^	10X	22 vs 12	24 vs 10	4.1 vs 4.4 *HR 0.98* *(0.73-1.31) P* *=* *.89*	4.8 vs 3.3 *HR 0.69* *(0.48-1.0) P* *=* *.049*	16.4 vs 14.0 *HR 0.82* *(0.58-0.15) P* *=* *.24*	17.3 vs 12.0^a^ *HR 0.71* *(0.49-1.02) P* *=* *.06*
	PS2+ (Exploratory)	NA	29 vs 6	NA	5.6 vs 3.2 *HR 0.54* *(0.33-0.89) P* *=* *.01*	NA	16.4 vs 11.4 *HR 0.67* *(0.41-1.19) P* *=* *.12*
**SORAYA** *Single arm (n* *=* *106)*^57^	PS2+	†	32	†	4.3	†	13.8
**MIRASOL** MIRV vs ICC *Phase III* (n = 453)^58^	PS2+	†	42 vs 16 *OR 3.81* *P* *=<* *.0001*	†	5.6 vs 3.9 *HR 0.65* *(0.52-0.81)* *P* *=<* *.0001*	†	16.4 vs 12.7 *HR 0.67* *(0.50-0.89)* *P* *=* *.0046*
**FORWARD II** *MIRV* *+* *BEV* *Phase Ib/II (n* *=* *94)*^59^	PS2 +	44	48	8.2	9.7	NA	NA

**PS2+ score**: ⩾25% of tumor cells with ⩾2 + staining intensity (low = 25%-50%, medium = 50%-74%, high ⩾ 75%).

**10X score**: ⩾50% of tumor cells with any FRα staining visible at ⩽10 microscope objective (medium = 50%-74%, high ⩾ 75%).

† All patients were FRα high.

a Final analysis.

Abbreviations: BEV, bevacizumab; ICC, investigator’s choice of chemotherapy; FRα, Folate receptor alpha; MIRV, Mirvetuximab soravtansine; mOS, median overall survival; mPFS, median progression-free survival; NA, not available; OR, odds ratio; ORR, overall response rate.

The FORWARD I trial, a randomized, phase III study was designed to evaluate the safety and clinical activity of MIRV as compared with the investigator’s choice of chemotherapy (ICC) in patients with FRα-positive PROC who had received 1 to 3 prior systemic lines of therapy. The study implemented a simplified scoring method to assess FRα expression (⩾50% of tumor cells with any FRα membrane staining visible at ⩽10× microscope objective was considered positive), centrally determined using the anti-FOLR1 2.1 antibody Ventana System.^60^ FRα expression criteria were changed from the initial phase 1/2 studies evaluating FRα as a predictive biomarker and leading to recommended phase 2 dose (RP2D), which used not only the proportion score but the intensity of membranous FRα staining, and only those samples with sufficient 2+ and 3+ intensity staining (PS2+ scoring) were considered positive.^33^

The primary endpoint was PFS, assessed by blinded-independent central review (BICR), in both the intention-to-treat population (ITT) and the high-FRα subgroup (⩾75% of tumor cells with any membrane staining). In the ITT population, 366 patients, there was no significant difference in PFS between groups (HR, 0.98, *P* = .897); median PFS was 4.1 and 4.4 months for MIRV and chemotherapy, respectively; there were no differences in overall survival (Table 1). In the prespecified high-FRα subgroup, PFS was longer in patients in the MIRV group compared with ICC (median, 4.8 vs 3.3 months; HR 0.69, *P* = .049), although not meeting statistical significance.^55^ ORR was higher in the MIRV group (24 vs 10%, *P* = .014), and there was a trend toward improvement in OS (17 vs 12 months, *P* = .063); secondary endpoints Ca-125 response, PFS-2, and patient-reported outcomes (PRO) all favored the MIRV group. MIRV was well tolerated, with fewer patients experiencing grade 3 drug-related adverse events (25% vs 44.0%), dose reductions (20% vs 30%), and discontinuations (4% vs 8%) compared with IC chemotherapy.

These unexpected results raised concern about the method used to determine FRα positivity. The assay used in Forward I was different from prior studies, and exploratory rescoring analyses using PS2+ methodology suggested that the use of <10× scoring allowed enrollment of patients with lower-than-expected levels of FRα expression, thus diluting the treatment effect of MIRV, and showed that patients with high FRα expression did have better outcomes^56^ (Table 1).

SORAYA was a single-arm trial (called phase III) designed to support accelerated approval in the United States. The study evaluated MIRV at 6 mg/kg every 3 weeks in 106 patients with PROC with high FRα expression by IHC (⩾75% of cells with PS2+ staining intensity), who had received 1 to 3 prior therapies, prior Bevacizumab was required. The primary endpoint of ORR assessed by the investigator (INV) was 32.4%, including 5 complete responses; the median duration of response (DOR) was 6.9 months, and the median PFS was 4.3 months^57^ (Table 1). Treatment-related adverse events (TRAE) led to dose delays in 32%, dose reductions in 19%, and discontinuations in 7% of patients.

Results of the SORAYA trial led to the Food and Drug Administration (FDA) accelerating the approval of MIRV on November 2022, as the first biomarker-directed therapy in platinum-resistant ovarian cancer.

Initial results from the confirmatory phase III randomized trial MIRASOL were presented at 2023 ASCO annual meeting. Four hundred fifty-three patients with FRα-high (PS + 2) PROC with 1 to 3 prior lines were randomized to MIRV 6 mg/kg adjusted ideal body weight or ICC: paclitaxel, PLD, or topotecan. Patients were a stratified number of prior systemic therapies and ICC; 61% received prior bevacizumab and 55% received prior PARPi. At a median follow-up of 13.1 months, MIRV demonstrated a statistically significant improvement in overall survival vs ICC (16.4 vs 12.75 months, HR 0.67, *P* = .0046); median PFS by the investigator was 5.62 vs 3.98 months (HR 0.65, *P* ⩽ .0001), and ORR was 42% with MIRV, compared with 16% with ICC (OR 3.81, *P* ⩽ .0001).^58^ Grade 3 + TRAE were reported in 42% of patients in the MIRV arm and in 52% in the ICC arm; discontinuation due to TRAE was 9% and 16%, respectively.

Recent results from the MIRASOL trial are expected to support the approval of MIRV in FRα-high PROC patients worldwide.

## Combination Strategies

The FORWARD II is a phase Ib/II trial (NCT 02606305) currently evaluating MIRV in combination with multiple agents, including Bevacizumab, Carboplatin, PLD, Pembrolizumab, or Bevacizumab plus Carboplatin, in patients with FRα-positive (PS2+ ⩾25%) relapsed ovarian cancer. Following a protocol amendment in November 2017, the threshold for FRα positivity was raised from ⩾25% to ⩾50% for continuing enrollment into the expansion cohorts due to evolving data on levels of FRα required for optimal efficacy, based on IMGN853-0401 first in the human study and initial results of FORWARD II.^61^

### MIRV plus bevacizumab

In the platinum-resistant setting, the addition of Bevacizumab to single-agent chemotherapy leads to an improvement in ORR and PFS as demonstrated in the pivotal AURELIA trial (ORR: 31% and median PFS = 6.7 months) that led to Bevacizumab approval for use alongside chemotherapy in this setting.^12^

The combination of MIRV and Bevacizumab was explored in the FORWARD II trial; the final analysis included 94 patients with platinum-resistant ovarian cancer, of which 88% had FRα-medium or high expression (PS2+ ⩾50%). The overall response rate was 44%, with a median DOR of 9.7 months and PFS of 8.2 months.^59^ The combination was safe and generally well tolerated; the summary of adverse events is shown in Table 2.

**Table 2.** Toxicity profile of MIRV + Bevacizumab.

Adverse event	FORWARD II^59^*(n* *=* *94)*	
	All grades (%)	Grade ⩾ 3 (%)
Blurred vision	57	1
Diarrhea	54	1
Nausea	51	1
Fatigue	43	3
Peripheral neuropathy	38	1
Keratopathy	34	0
Thrombocytopenia	30	4
Decreased appetite	28	0
Hypertension	28	15
Dry eye	28	2
AST increase	27	4
Vomiting	27	1
Epistaxis	22	1

Results in the cohort of platinum-agnostic patients (47% were platinum-sensitive and 53% platinum-resistant) demonstrated an ORR of 47% and median PFS 8.3 months. Interestingly, in patients with high FRα expression (PS2+ ⩾75%), ORR was 59% in the platinum-resistant and 69% in the platinum-sensitive population.^62^

An updated analysis from the phase Ib/II study was recently presented at the 2022 International Gynecologic Cancer Society annual meeting. Among 126 heavily pretreated patients (46% ⩾ 3 prior therapies, 75% platinum-resistant and 52% prior Bevacizumab), ORR was 44% and median PFS 8.2 months.^63^ Of note, ORR was 58% in the Bevacizumab-naive population and 32% in prior Bevacizumab exposure.

These results showed a high level of activity in patients with high-FRα-positive tumors, independent of platinum status, and even with prior Bevacizumab exposure. MIRV also showed promising activity in platinum-sensitive disease and can be considered in patients in whom a nonplatinum doublet would be appropriate (eg, hypersensitivity, excessive risk of toxicity). In the platinum-resistant setting, the ORR of MIRV and Bevacizumab was 59% in this trial, compared with the expected 50% for the standard Paclitaxel and Bevacizumab; these results warrant further investigation and face-to-face comparison between these combinations.

### MIRV plus chemotherapy

As part of the aforementioned FORWARD II study, MIRV was evaluated in combination with Carboplatin in the platinum-sensitive setting. Results were presented at ESMO 2020 Congress. Forty-one patients with FRα positivity (medium/high expression, PS2+ ⩾50%) and 1 to 2 prior lines of therapy received MIRV with Carboplatin (AUC 5) and Bevacizumab (15 mg/kg) every 21 days; MIRV and Bevacizumab were continued as maintenance after completing Carboplatin; 42% received previous PARPi and 24% had prior Bevacizumab. Confirmed ORR was 81%, with median DOR of 10.7 months and a median PFS of 12 months.^64^

Responses with platinum doublet chemotherapy plus Bevacizumab in the platinum-sensitive relapse are reported to be around 70% to 80% and PFS between 12 and 14 months.^14,65^ MIRV plus Carboplatin appears a promising combination as an alternative to current treatment regimens, but further confirmation in larger clinical trials is warranted. The MIROVA clinical trial (NCT04274426) is currently evaluating Carboplatin plus MIRV vs standard platinum-based chemotherapy in this setting.^66^

The combination of MIRV and PLD was explored in the dose-escalation phase of the FORWARD II trial. The combination was safe, the highest dose level evaluated was 6.0 mg/kg of MIRV and 40 mg/kg of PLD administered on day 1 of a 4-week cycle (n = 16).^67^ Toxicity was manageable, and the most frequent adverse events were diarrhea (56%), constipation (50%), and fatigue and nausea (each 44%); no dose-limiting toxicities (DLTs) were observed. Efficacy data have not yet been published.

### MIRV plus immunotherapy

Preliminary data from the dose-escalation phase of the FORWARD II trial, reported clinical activity of MIRV plus Pembrolizumab. Among 14 patients with PROC with FRα positivity (PS2+ ⩾25%) and 2 to 4 prior lines of systemic therapy, the combination demonstrated favorable tolerability, with primarily ⩽ grade 2 adverse events observed, and resulted in an ORR of 43%, median DOR of 6.9 months, and median PFS of 5.2 months.^68^ To confirm this preliminary data, the expansion phase of this arm is currently in progress.

## Toxicity

The main TRAE of MIRV monotherapy reported in the initial phase I^51^ and 2 phase III trials^55,57^ include blurred vision (41%-42%), nausea (29%-46%), diarrhea (22%-51%), fatigue (24%-30%), and peripheral neuropathy (13%-28%); most of ⩾grade 3 events occurred in less than 5%. A summary of adverse events (AEs) across trials is presented in Table 3.

**Table 3.** Toxicity profile of MIRV monotherapy across different clinical trials.

Adverse event	IMGN853-0401^51^ *(n* *=* *46)*		SORAYA^57^ *(n* = *106)*		FORWARD I^55^ *(n* *=* *243)*		MIRASOL^58^ *(n* *=* *453)*	
	All grades (%)	Grade ⩾ 3 (%)	All grades (%)	Grade ⩾ 3 (%)	All grades (%)	Grade ⩾ 3 (%)	All grades (%)	Grade ⩾ 3 (%)
Diarrhea	43	2.2	22	2	31	2.1	29	1
Blurred vision	41	0	41	6	42	2.5	41	8
Nausea	37	2.2	29	0	46	1.2	27	2
Fatigue	30	4.3	24	1	29	1.2	NA	NA
Peripheral neuropathy	28	2.2	13	0	27	2.5	22	1
Dry eye	13	0	25	2	26	1.2	28	3
AST increase	24	2.2	NA	NA	16	1.2	NA	NA
Decreased appetite	NA	NA	13	1	17	0.8	NA	NA
Vomiting	22	2.2	11	0	16	1.2	NA	NA
Keratopathy	26	0	29	9	32	1.2	32	9
Neutropenia	NA	NA	13	2	6.6	0	11	1
Asthenia	NA	NA	15	1	18	0.8	NA	NA
Anemia	13	2.2	NA	NA	11	0.8	10	1

Abbreviations: NA, not available.

Ocular AE such as blurred vision and keratopathy represent an important clinical concern for molecularly targeted therapies.^69,70^ Corneal toxicity is nonreceptor-mediated, evidenced by a lack of FRα expression in human corneal tissues and preclinical modeling studies in rabbits, but rather mediated by the antimitotic activity of the DM4 payload.^71^

In the initial phase I study of MIRV, ocular abnormalities emerged as an AE of interest during the escalation phase, triggering dose modifications^50^; they were generally mild.^72^Correlation between ocular events with dose and exposure resulted in the modification of MIRV dosing from total to adjusted ideal body weight to decrease the drug exposure variance between patients.^50^ In addition, implementation of daily lubricating eye drops and other proactive measures such as avoidance of contact lenses and application of compresses over the eyes subsequently decreased the incidence and grade of patients’ visual disturbances during treatment. Steroid eye drops have also proven useful to manage ocular symptoms, and as primary and secondary prophylaxis.^51,71^

## Future Investigations

With the encouraging clinical results that led to FDA approval of MIRV for FRα-positive, PROC, investigations are now shifting to earlier settings, where MIRV is being explored both in monotherapy and in combination with other agents.

In the platinum-resistant setting, phase I studies are evaluating the combination of MIRV with novel agents, such as the bifunctional fusion protein CD47/CD40 SL-172154. In the platinum-sensitive relapse, MIRV is currently being explored as monotherapy in the PICCOLO study for patients who cannot receive or tolerate rechallenge with platinum, and in combination with Carboplatin vs standard of care in the MIROVA trial. MIRV is also being studied as a maintenance strategy in combination with Bevacizumab in the GLORIOSA study and as a neoadjuvant treatment in combination with Carboplatin in a phase II trial.

Table 4 shows undergoing clinical trials evaluating novel strategies, including MIRV both in the first line and in the recurrence setting.

**Table 4.** Ongoing clinical trials investigating MIRV in ovarian cancer.

NCT Number	Study name	Phase	Setting	Intervention	Status
NCT04606914		II	Neoadjuvant	• Carboplatin plus Mirvetuximab soravtansine	Recruiting
NCT05041257	PICCOLO	II	Platinum-sensitive	• Mirvetuximab soravtansine monotherapy	Recruiting
NCT04274426	MIROVA	II	Platinum-sensitive	• Platinum-based chemotherapy (Carboplatin plus: PLD, Gemcitabine or Paclitaxel). • Carboplatin plus Mirvetuximab soravtansine x 6 cycles, followed by Mirvetuximab soravtansine maintenance	Recruiting
NCT05456685	IMGN853-0420	II	Platinum-sensitive	• Carboplatin plus Mirvetuximab soravtansine x 6 cycles, followed by Mirvetuximab soravtansine maintenance if no progression	Recruiting
NCT05445778	GLORIOSA	III	Platinum-sensitive maintenance	• Mirvetuximab soravtansine plus Bevacizumab • Bevacizumab alone	Recruiting
NCT05483933	SL03-OHD-105	I	Platinum-resistant	SL-172154 plus: • Mirvetuximab soravtansine, or • PLD	Recruiting
NCT03552471		I	Relapsed ovarian/endometrial	• Mirvetuximab soravtansine plus Rucaparib camstylate	Active, not recruiting
NCT02996825		I	Relapsed ovarian/endometrial/TNBC	• Mirvetuximab soravtansine plus Gemcitabine	Active, not recruiting

Abbreviations: PLD, pegylated liposomal doxorubicin; TNBC, triple-negative breast cancer.

## MIRV in Non-HGSOC and Endometrial Cancer

Differential levels of FRα expression have been observed across different histological subtypes of ovarian cancer. Data from a consortium-based study including 2801 patients reported FRα expression in 76% of high-grade serous, 49% of low-grade serous, and 32% of clear-cell ovarian cancers.^73^ In this study, patients with FRα-positive clear cell carcinomas (CCC) showed decreased PFS independent of follow-up time (HR = 1.89, 95% CI = 1.10-3.25, N = 259). It remains to be confirmed whether FRα-directed therapies improve clinical outcomes in patients with non-HGSOC.

FRα is expressed in 20% to 50% of endometrial tumors^74^ and has been associated with poor prognostic factors including high-grade, advanced-stage, and nonendometrioid histology.^75^ In a phase I study of 24 patients with recurrent endometrial cancer (EC), 67% uterine serous carcinomas (USC), MIRV was associated with 2 confirmed partial responses and 11 stable diseases, with an overall clinical benefit rate >50% (NCT01609556). Another phase I study evaluating MIRV plus Rucaparib in 18 heavily pretreated patients with FRα-positive EC and ovarian cancer found that the combination was well tolerated, with and ORR of 43%; among five patients with EC, ORR was 50% and mPFS was 6.6 months (95% CI, 0.5-11.9).^76^ Currently, a phase II trial is exploring MIRV plus Pembrolizumab in patients with microsatellite stable (MSS) and FRα-positive advanced/recurrent USC.^77^

## New Directions in ADC Development

New-generation ADCs have optimal specificity and cytotoxicity profiles. Nevertheless, there remain many challenges in their development, including complexity in pharmacokinetics, insufficient tumor targeting and payload release, as well as drug resistance.^78^

ADC consists of a monoclonal antibody, linker, and payload; its modular nature enables each component to be changed or altered in a strategic fashion.^17^ For ADCs to have better safety and efficacy profile, there are many factors that might be optimized.

First, enhancing specificity, affinity, and pharmacokinetics are of great importance for the optimization of therapeutic mAbs.^18^ Different antibodies targeting the same antigen may have different binding abilities and different effects on receptor dimerization and antigen internalization, which may have marked effects on their in vivo activity.^79^ Also, targeting mutant proteins with ADCs can potentially lead to improved tumor response, as they usually have higher ubiquitination levels and are easier to be internalized and degraded.^80^

Design of bispecific antibodies containing 2 different antigen-binding sites may improve antibody internalization and improve tumor specificity.^81^ Andreev and colleagues generated a bispecific antibody–based ADC that was able to bind to HER2 and prolactin receptor (PRLR) expressed on breast cancer cells.^82^ They compared the effects of different concentrations of HER2 ADC, HER2/PRLR bispecific ADC, and PRLR ADC on the viability of BT-483 cells, which express 6.90 × 104 HER2 surface receptors per cell (intermediate-to-low HER2) and approximately 8.0 × 103 PRLR surface receptors per cell. HER2 ADC and PRLR ADC had relatively modest effects on BT-483 cell viability (1.5 and 2.5 nM IC_50_, respectively); in contrast, the killing effect was dramatically increased when cells were treated with HER2/PRLR ADC judging by the reduced percentage of viable cells at lower ADC concentrations. HER2 ADC + PRLR ADC combination (0.4 nM IC_50_) also increased cell killing in comparison with HER2 ADC or PRLR ADC alone, but to a lesser extent than HER2/PRLR bispecific ADC (0.15 nM IC_50_).^82^

ADCs built on biparatopic antibodies, which target 2 separate epitopes of the same target antigen, can induce receptor clustering and rapid target internalization.^83^ Similarly, a dual-payload ADC that employs 2 distinct synergistic cytotoxic agents as payloads could be developed to achieve a greater efficacy and reduce drug resistance.^84^

Another strategy currently being explored is the optimization of payloads. One of the new payloads used in ADCs design is pyrrolobenzodiazepines (PBDs), which are sequence-selective DNA-alkylating compounds produced by several actinomycetes.^85^ PBDs bind and cross-link with a specific target of cancer cell DNA, preventing tumor cells multiplication without deforming its DNA helix, thus potentially avoiding the emergence of drug resistance.^86^ Amatoxin, spliceostatin C, and thailanstatin A are other new payloads that work as RNA polymerase inhibitors.^87^ The choice of payload is moving beyond standard cytotoxic drugs to targeted and immunotherapeutic agents. Mirzotamab clezutoclax is a B7H3 (CD276)-targeted ADC carrying a pro-apoptotic BCL-XL inhibitor payload, currently being investigated in early-phase clinical trials.^88,89^

Finally, another ADC development strategy is to abandon the traditional structure of mAb and couple the payload to the polypeptide fragment or single-chain variable region fragment, to reduce the molecular weight of ADCs and improve penetration efficiency as well as payload delivery; for example, PEN-221, a 2-kDa peptide-DM1 conjugate targeting somatostatin receptor 2 (SSTR2) is being investigated for the treatment of neuroendocrine tumors and small-cell lung cancer in a phase 1/2a clinical trial (NCT02936323). The potential challenge of small-molecule drug conjugates is that they can be rapidly cleared in plasma.^90^

## Conclusions

Intrinsic and acquired platinum resistance is associated with a dismal prognosis and represents an unmet clinical challenge. Available systemic therapies in this setting have limited efficacy and considerable toxicity. With our evolving understanding of tumor biology and advances in drug development, ADCs have shown potential to overcome these obstacles by selectively delivering cytotoxic drugs into tumor cells, enhancing activity, and decreasing systemic toxicity.

FRα is aberrantly expressed in the majority of epithelial ovarian cancers and minimally expressed in normal tissues; therefore, it is fit for purpose as a therapeutic/theranostic target, and serves as a predictive biomarker for targeted strategies such as ADCs, designed to exploit this differential distribution pattern.

MIRV is a first-in-class ADC that has been shown to improve clinical outcomes in patients with FRα-positive platinum-resistant ovarian cancer and has been included in the treatment paradigm. Preliminary data report a robust clinical activity in combination with Bevacizumab in both the platinum-sensitive and -resistant settings. Moreover, studies are now evaluating its role in combination with established and novel therapies as well as maintenance and neoadjuvant strategies.

The field of theranostics is evolving rapidly and the initial successes with receptor-directed ADCs would allow deeper exploration and integration of precision-targeted cytotoxic agents with immunotherapy.
